# Metabolic and cardiorespiratory effects of decreasing lung hyperinflation with budesonide/formoterol in COPD: a randomized, double-crossover, placebo-controlled, multicenter trial

**DOI:** 10.1186/s12931-020-1288-3

**Published:** 2020-01-20

**Authors:** Miguel J. Divo, Michael R. DePietro, John R. Horton, Cherie A. Maguire, Bartolome R. Celli

**Affiliations:** 1 Pulmonary and Critical Care Division, Brigham and Women’s Hospital, Harvard Medical School, 75 Francis Street, Boston, MA 02115 USA; 2 AstraZeneca LP, Wilmington, DE USA; 3 Current affiliation: Teva Pharmaceuticals, Frazer, PA USA

**Keywords:** Oxygen uptake, Cardiac output, Oxygen pulse, Stroke volume

## Abstract

**Background:**

Studies suggest that acute decreases in lung hyperinflation at rest improves cardiac function and increases lung vascular perfusion from decompression of a compromised heart. In those studies, changes in resting oxygen uptake induced by medications, an alternative explanation for compensatory increased cardiac function, were not explored.

**Methods:**

This double-blind, multicenter, double-crossover study enrolled adults with chronic obstructive pulmonary disease, resting hyperinflation, and > 10% improvement in inspiratory capacity after 2 inhalations of budesonide/formoterol 160/4.5 μg. Metabolic, cardiac, and ventilatory function were measured 60 min pre−/post-dose at each visit. Primary endpoint was change in resting oxygen uptake for budesonide/formoterol versus placebo.

**Results:**

Fifty-one patients (median age: 63 years) received treatment. Compared with placebo, budesonide/formoterol significantly increased resting oxygen uptake (mean change from baseline: 1.25 vs 11.37 mL/min; *P* = 0.007) as well as tidal volume and minute ventilation. This occurred despite improvements in the inspiratory capacity, forced vital capacity, and expiratory volume in 1 s. No significant treatment differences were seen for oxygen saturation, respiratory rate, and resting dyspnea. There was a numerical increase in oxygen pulse (oxygen uptake/heart rate). Correlations between inspiratory capacity and oxygen pulse were weak.

**Conclusions:**

Budesonide/formoterol treatment in resting hyperinflated patients with COPD results in significant deflation. The increase in oxygen uptake and minute ventilation at lower lung volumes, without changes in heart rate and with minimal improvement in oxygen pulse, suggests increased oxygen demand as a contributor to increased cardiac function.

**Trial registration:**

**ClinicalTrials.gov** **identifier:** NCT02533505.

## Background

Patients with moderate-to-severe chronic obstructive pulmonary disease (COPD) are likely to have static lung hyperinflation, which confers a poor prognosis [1]. Resting hyperinflation is easily detected by measuring lung volumes during standard pulmonary function testing [2]. Determination of inspiratory capacity (IC) as a reflection of the end-expiratory lung volume at rest and during exercise has been shown to be a reliable, easy-to-measure, and practical variable to determine the degree of static and dynamic hyperinflation [3, 4]. Treatment with inhaled bronchodilators with or without corticosteroids decreases lung hyperinflation, and increases IC, which relates well to improvement in exercise endurance and dyspnea in these patients [5–9].

Hyperinflation has been linked to low cardiac output in patients with COPD [10], in part by limiting left ventricular stroke volume [11, 12]. Reversing hyperinflation through lung volume reduction surgery improves cardiac function at rest and during exercise [13, 14]. Measuring the oxygen pulse, obtained by dividing the measured resting oxygen uptake (VO_2_) by the heart rate (HR), provides an adequate reflection of cardiac stroke volume when the systemic extraction of oxygen is stable [12]. This method has been used to evaluate the effect of static and dynamic hyperinflation on cardiac function during exercise [13].

Whereas significant knowledge exists about the interaction between dynamic acute lung hyperinflation and cardiac function during exercise [7, 10, 12], only 2 studies have evaluated the effect of pharmacological decrease of hyperinflation on pulmonary tests and cardiac function at rest in patients with COPD [15–17]. Using magnetic resonance imaging (MRI) to measure cardiac chamber volume and function at rest, the study by Stone et al. showed that 1 week of once-daily inhaled fluticasone furoate/vilanterol (an inhaled corticosteroid [ICS]/long-acting beta_2_-agonist [LABA]) decreased resting lung volumes and increased right ventricular end-diastolic volume index, as well as cardiac index, without changes in intrinsic cardiac function [15]. Similar findings were reported in the second study, which used a combination of inhaled dual bronchodilators containing the LABA indacaterol plus the long-acting muscarinic antagonist (LAMA) glycopyrronium, administered over 2 weeks [16, 17]. In those studies, the authors attributed the improvement of heart function and increased pulmonary vascularity to an increase in cardiac volume resulting from lung deflation and associated decompression of the heart. However, no measurements were made of other factors that may contribute to an increase in cardiac demands, such as the increase in resting VO_2_ that occurs with administration of inhaled beta-agonists, as these agents have been shown to increase the metabolic demand of peripheral muscles [18, 19]. Interestingly, those studies did not find a relationship between lung function, including changes in IC and the improvement in cardiac function, suggesting the presence of other mechanisms to account for the increase in cardiac function. To our knowledge, no study has evaluated the metabolic function and dynamic ventilatory response in hyperinflated patients with COPD after decreasing resting lung volumes acutely with inhaled pharmacotherapy that includes beta-agonists.

The aim of this study of patients with COPD and resting hyperinflation, therefore, was to test the hypothesis that a single dose of inhaled budesonide/formoterol (administered at 2 different visits), could alter resting metabolic demand (VO_2_) while decreasing resting lung volumes. The impact on cardiac and respiratory function in these patients while at rest was also examined. Inhaled placebo randomly administered in the separate visits served as control.

## Methods

### Study design and patient selection

**Fig. 1:**
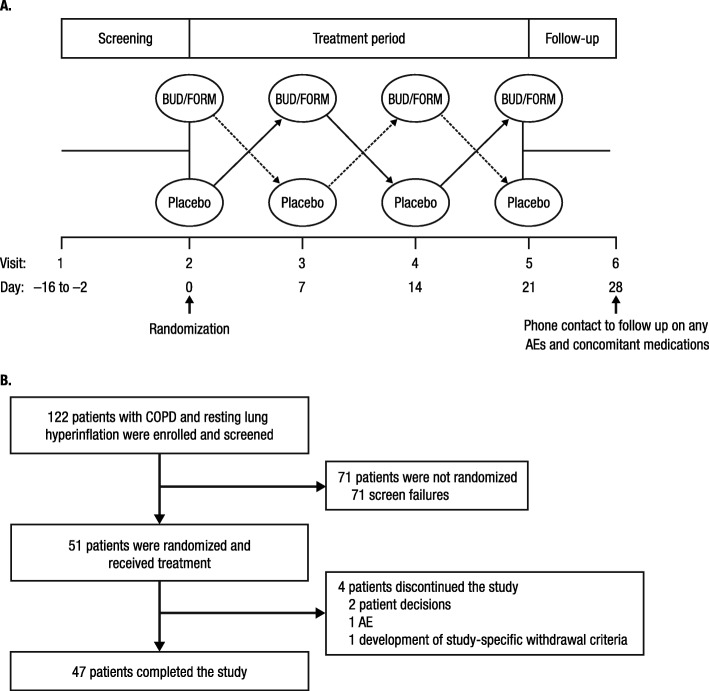
Summary of (**a**) study design and (**b**) patient disposition. AEs, adverse events; BUD/FORM, budesonide/formoterol; COPD, chronic obstructive pulmonary disease; pMDI, pressurized metered-dose inhaler

This randomized, double-blind, multicenter, placebo-controlled, double-crossover study included a screening visit, 4 treatment visits 7 days apart, and 1 follow-up telephone visit (Fig. 1a). Eligible patients were aged 40 to 80 years (inclusive) with a clinical diagnosis of COPD, post-bronchodilator forced expiratory volume in 1 s (FEV_1_)/forced vital capacity (FVC) ratio <  0.7, and post-bronchodilator FEV_1_ ≤ 65% of predicted. Lung hyperinflation was defined as an increase in IC of > 10% after 2 inhalations of open-label budesonide/formoterol 160/4.5 μg (total dosage 320/9.0 μg; Symbicort®; AstraZeneca Pharmaceuticals LP, Wilmington, DE, USA) from a pressurized metered-dose inhaler (pMDI), administered with a spacer at screening. All patients were in clinically stable condition, per the complete inclusion/exclusion criteria (Additional file 1). The study was approved by the local ethics review committee (Additional file 1: Table S1) and conducted in accordance with the Declaration of Helsinki; all patients provided written informed consent.

Following current medication washout, patients randomly received 1 dose of 2 inhalations of budesonide/formoterol 160/4.5 μg from a pMDI or matching placebo with a BreatheRite® spacer.

### Physiologic measurements

Measurements were taken 60 min pre-dose and 60 min post-dose for each visit. Gas exchange and respiratory variables measured at rest using a metabolic cart with cardiac monitoring were resting VO_2_, resting carbon dioxide output (VCO_2_), respiratory rate (RR), tidal volume (V_T_), inspiratory time (T_i_), expiratory time, oxygen saturation, and HR; calculated variables were oxygen pulse (VO_2_/HR), inspiratory flow rate (IFR;: V_T_/T_i_), total respiratory time (60/RR), respiratory time fraction (T_i_/total respiratory time), and minute ventilation (V_e_; :RR × V_T_). Sitting systolic and diastolic blood pressure were also measured during screening and at each visit. To assess pulmonary function, total lung capacity, functional residual capacity, residual volume, and slow vital capacity (SVC) were measured with body plethysmography during screening, and FEV_1_, FVC, and IC were measured using spirometry at each visit (post-screening IC was measured using an SVC maneuver). Dyspnea was scored at each treatment visit using the Modified Borg Dyspnea Scale. See Additional file 1 for full details.

### Statistical analyses

Statistical analysis was performed using SAS® version 9.4. The efficacy analysis set included all randomized patients who completed ≥1 post-baseline measurement of the primary efficacy endpoint under each of the 2 treatment groups; the safety analysis set included all patients who received ≥1 dose of randomized study medication. Each patient received 2 placebo and 2 active medication treatments (Fig. 1a). Treatment group estimates were provided as least squares means. The primary efficacy endpoint was change from pre-dose to post-dose assessment in resting VO_2_ after administration of budesonide/formoterol versus placebo (measured at the 4 post-baseline visits). A restricted maximum likelihood–based mixed model for repeated measures was employed using sequence, treatment, and visit as fixed effects, with patient nested within sequence as a random effect. See Additional file 1 for secondary efficacy measures. *P* values with estimated treatment differences and 95% confidence intervals were calculated for efficacy comparisons. Associations between primary and secondary efficacy endpoints for each treatment group were computed using the Pearson correlation matrix across all treatment visits.

## Results

A total of 122 patients were screened, and 51 patients were randomized (Fig. 1b) and included in both the efficacy and safety analyses (first patient was enrolled on August 27, 2015; last patient completed the study on August 12, 2016). The demographic and clinical characteristics of the patients who completed the study are summarized in Table 1. The population was 47% women with a mean (standard deviation) age of 62.9 (8.32) years. Consistent with the inclusion criteria, patients had moderate to severe airflow limitation and hyperinflation.

**Table 1:** Demographic and clinical characteristics

Characteristics	Total (*N* = 51)
Demographic^a^	
Age, mean (SD), y	62.9 (8.32)
Women, n (%)	24 (47)
BMI, mean (SD), kg/m^2^	28.02 (6.88)
Race, n (%)	
White	37 (72.5)
African American	13 (25.5)
Other	1 (2.0)
Clinical^b,c^	
IC, L	1.934 (0.526)
Change after administration of BUD/FORM	0.367 (0.169)
FEV_1_, L	1.147 (0.366)
Change after administration of BUD/FORM	0.191 (0.114)
FVC, L	2.497 (0.802)
Change after administration of BUD/FORM	0.308 (0.218)
FEV_1_/FVC	0.472 (0.097)
Change after administration of BUD/FORM	0.017 (0.036)
TLC, L	6.214 (1.221)
FRC, L	4.314 (1.018)

*BMI* body mass index, *BUD/FORM* budesonide/formoterol, *FEV*_*1*_ forced expiratory volume in 1 s, *FRC* functional residual capacity, *FVC* forced vital capacity, *IC* inspiratory capacity, *SD* standard deviation, *TLC* total lung capacity

^a^At baseline

^b^At screening

^c^All values reported as mean (SD)

### Gas exchange and cardiac parameters

**Fig. 2:**
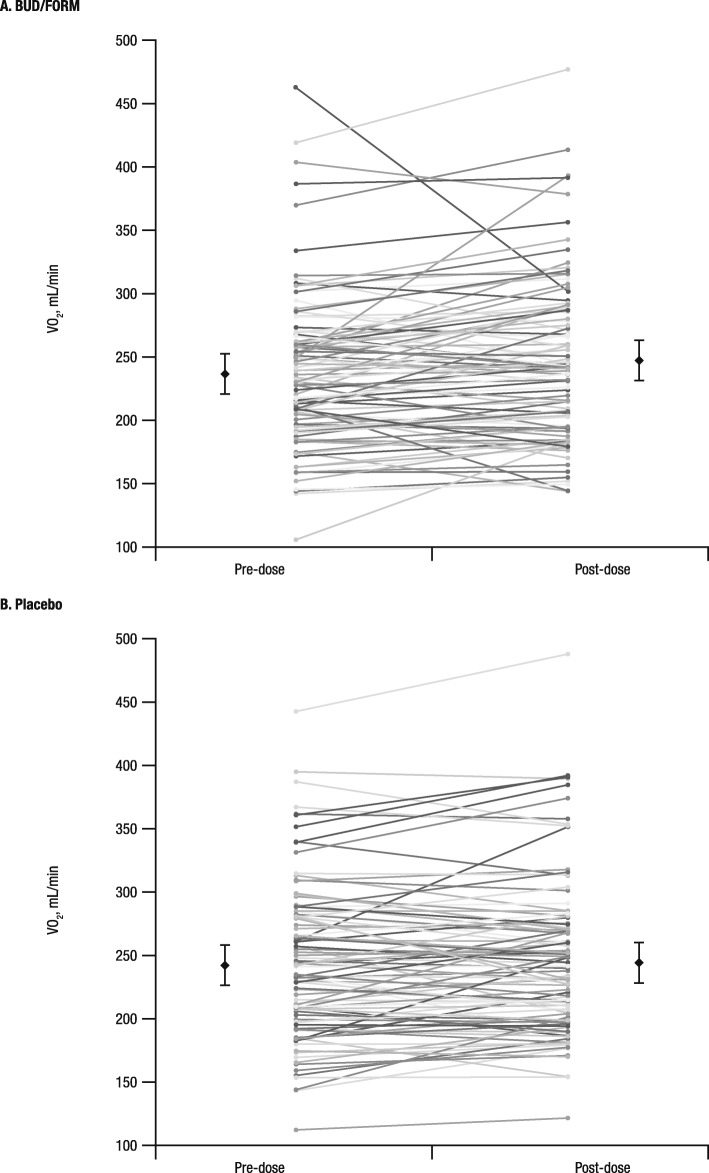
Oxygen uptake pre-dose and post-dose for patients after administration of (**a**) BUD/FORM and (**b**) placebo.^*,†^. BUD/FORM, budesonide/formoterol; CI, confidence interval; LS, least squares; VO_2_, oxygen uptake. ^*^Each line represents an individual patient and treatment. ^†^In each panel, the pair of data points with error bars are LS mean (95% CI) values at pre-dose and post-dose

Resting VO_2_ increased significantly after budesonide/formoterol treatment compared with placebo (11.37 vs 1.25 mL/min, respectively; *P* = 0.007; Table 2). Resting VO_2_ values for individual patients before and after treatment with budesonide/formoterol and placebo are shown in Fig. 2a and b, respectively. The observed increase in resting VO_2_ was associated with a significant increase in resting VCO_2_ with budesonide/formoterol compared with placebo.

**Table 2:** Differences in metabolic and cardiac variables before and after administration of BUD/FORM or placebo and comparison of the change between treatment groups

Outcome, unit	LS mean change from pre-dose to post-dose		LS mean (95% CI) treatment difference	*P* value^*^
	BUD/FORM	Placebo		
VO_2_, mL/min	11.37	1.25	10.11 (2.94 to 17.29)	**0.007**
HR, bpm	−2.48	− 2.83	0.35 (−1.02 to 1.72)	0.609
VO_2_/HR, mL/beat	0.256	0.168	0.087 (− 0.021 to 0.196)	0.111
VCO_2_, mL/min	5.99	−4.25	10.25 (2.47 to 18.02)	**0.011**
SaO_2_, %	0.42	0.18	0.24 (−0.26 to 0.74)	0.333

*bpm* beats per minute, *BUD/FORM* budesonide/formoterol, *CI* confidence interval, *HR* heart rate, *LS* least squares, *SaO*_*2*_ oxygen saturation, *VCO*_*2*_ carbon dioxide output, *VO*_*2*_ oxygen uptake, *VO*_*2*_*/HR* oxygen pulse

^*^Bolded *P* values are statistically significant

(5.99 vs − 4.25 mL/min, respectively; *P* = 0.011). Although there was no change in HR in either group, there was a non-significant trend toward an increase in oxygen pulse after patients received budesonide/formoterol (Table 2).

### Pulmonary function, respiratory parameters, dyspnea

Significant improvements were observed with budesonide/formoterol compared with placebo for IC (0.256 vs − 0.024 L; *P* <  0.001), FEV_1_ (0.187 vs − 0.004 L; *P* <  0.001), FVC, and FEV_1_/FVC (Table 3). V_e_ also increased significantly after the use of budesonide/formoterol compared with placebo. This difference is explained by an increase in V_T_ and no change in RR with budesonide/formoterol. In addition, there was a significant increase in the mean IFR (V_T_/T_i_) after the use of budesonide/formoterol compared with placebo (26.53 vs 3.22 mL/sec; *P* = 0.021). There were no differences between treatments for respiratory time fraction (T_i_/total respiratory time) or RR.

**Table 3:** Differences in respiratory function parameters before and after administration of BUD/FORM or placebo and comparison of the change between treatment groups

Outcome, unit	LS mean change from pre-dose to post-dose		LS mean (95% CI) treatment difference	*P* value^*^
	BUD/FORM	Placebo		
IC, L	0.256	− 0.024	0.280 (0.218 to 0.342)	**< 0.001**
FEV_1_, L	0.187	− 0.004	0.191 (0.150 to 0.233)	**< 0.001**
FVC, L	0.259	−0.052	0.312 (0.236 to 0.388)	**< 0.001**
FEV_1_/FVC	0.017	−0.002	0.019 (0.005 to 0.033)	**0.007**
V_T_, mL	71.90	14.28	57.62 (29.70 to 85.55)	**< 0.001**
V_T_/T_i_, mL/sec	26.53	3.22	23.32 (3.72 to 42.91)	**0.021**
T_i_/T_tot_	0.012	−0.004	0.016 (−0.004 to 0.036)	0.113
RR, breaths/min	−0.19	−0.43	0.24 (− 0.44 to 0.91)	0.484
V_e_ (RR × V_T_), mL/min	838	−23.9	862 (440 to 1284)	**< 0.001**
MBS^a^	−0.45	−0.25	− 0.20 (− 0.45 to 0.05)	0.106

*BUD/FORM* budesonide/formoterol, *CI* confidence interval, *FEV*_*1*_ forced expiratory volume in 1 s, *FVC* forced vital capacity, *IC* inspiratory capacity, *LS* least squares, *MBS* Modified Borg Dyspnea Scale, *RR* respiratory rate, *T*_*i*_*/T*_*tot*_ respiratory time fraction, *V*_*e*_ minute ventilation, *V*_*T*_ tidal volume, *V*_*T*_*/T*_*i*_ inspiratory flow rate

^a^Dyspnea was scored using the MBS, in which patients were asked to report their perception of breathing difficulty using a scale ranging from 0 (nothing at all) to 10 (extremely strong/maximal)

^*^Bolded *P* values are statistically significant

Mean changes in the Modified Borg Dyspnea Scale showed greater numerical improvement with the use of single-dose budesonide/formoterol, with no significant difference compared with placebo (Table 3).

### Correlation between changes in IC and other outcomes

Correlations between lung hyperinflation, as measured by change in IC, and changes in other primary and secondary outcomes were weak, with correlation coefficients ranging from an absolute value of 0.016 (IC with IFR [V_T_/T_i_]) to 0.522 (IC with FVC; Table 4).

**Table 4:** Correlations between change in IC and selected respiratory and cardiac variables for BUD/FORM and placebo

Outcome, unit	Pearson correlation coefficient (vs IC)^a^
VO_2_, mL/min	0.067
HR, bpm	−0.079
VO_2_/HR, mL/beat	0.138
VCO_2_, mL/min	0.022
SaO_2_, %	0.115
FEV_1_, L	0.432
FVC, L	0.522
FEV_1_/FVC	0.126
V_T_, mL	0.191
V_T_/T_i_, mL/sec	−0.016
T_i_/T_tot_	0.181
RR, breaths/min	−0.063
V_e_ (RR × V_T_), mL/min	0.158
MBS	−0.051

bpm, beats per minute; BUD/FORM, budesonide/formoterol; FEV_1_, forced expiratory volume in 1 s; FVC, forced vital capacity; HR, heart rate; IC, inspiratory capacity; MBS, Modified Borg Dyspnea Scale; RR, respiratory rate; SaO_2_, oxygen saturation; T_i_/T_tot_, respiratory time fraction; VCO_2_, carbon dioxide output; V_e_, minute ventilation; VO_2_, oxygen uptake; VO_2_/HR, oxygen pulse; V_T_, tidal volume; V_T_/T_i_, inspiratory flow rate

^a^Pearson correlation computed between the given efficacy endpoints (change from pre-dose to post-dose) for BUD/FORM and placebo across all visits

### Safety

Adverse events (AEs) were reported in a similar proportion of patients after treatment with budesonide/formoterol (26%) and placebo (22%; Additional file 1: Table S2). After budesonide/formoterol treatment, there were no serious AEs, AEs leading to discontinuation, or causally related AEs. After placebo, there was 1 serious AE (pneumonia), 2 AEs leading to discontinuation (COPD exacerbation, chronic bronchitis exacerbation), and 1 causally related AE (headache). There were no deaths during the study.

## Discussion

This study of patients with COPD with lung hyperinflation at rest demonstrated that single-dose administration of 2 inhalations of budesonide/formoterol 160/4.5 μg (total dosage 320/9.0 μg) decreased resting lung hyperinflation. Despite this seemingly beneficial effect on respiratory mechanics, there was a significant increase in resting VO_2_ and resting VCO_2_ with concomitant increases in minute ventilation compared with placebo. The increase in cardiac function after lung deflation in this and other studies with beta-agonist–containing medications may in part be the response to an increase in metabolic demand rather than just better heart function secondary to the improvement in ventilatory mechanics due to lung deflation.

The most novel and clinically relevant findings in this study are the significant increases in resting VO_2_ and resting VCO_2_ observed after single-dose inhalation of budesonide/formoterol. Because this finding was observed in a double-blind, double-crossover, multicenter design study of patients at rest, it cannot be related to augmented physical activity. The increases in resting VO_2_ and resting VCO_2_, despite a decrease in resting lung volume as indicated by improvements in airflow limitation and IC, as well as an improvement in all spirometric parameters (FVC, FEV_1_, and FEV_1_/FVC) were surprising because those changes are associated with decreased work of breathing. The improvement in respiratory mechanics secondary to the deflation should have resulted in either no change or a decrease in oxygen uptake. The findings of an *increase* in VO_2_ at rest are most consistent with an increase in peripheral muscle utilization of oxygen with increased oxygen extraction, as the changes in cardiac function were minimal. Data from previous studies using the beta-agonist salbutamol support this finding [20]. This appears to be a function of all beta-agonists. Indeed, it has been shown that infusion of the beta-agonist epinephrine activates various glycolytic enzymes and elevates carbohydrate oxidation and glycogen utilization in skeletal muscles [21, 22]. It could be argued that epinephrine is a non-selective agonist with affinity for both alpha- and beta-adrenoceptors, which may not have the same effect as more selective beta-agonists. However, studies with more selective beta_2_-agonists including formoterol (as was used in this study) have shown an increase in systemic concentrations of plasma lactate in exercising humans, which suggests a stimulatory action on glycolysis of working skeletal muscles [18–20, 23–25]. The increase in V_T_ and V_e_ observed in this study despite improved lung mechanics are consistent with an increased respiratory response to match the peripheral oxygen uptake increase, or from direct central respiratory drive stimulus as has been shown in healthy individuals given intravenous salbutamol [24].

Interestingly, very few studies have evaluated the acute effect of inhaled beta-agonists on respiratory and cardiac function at rest in patients with COPD, even though inhaled beta_2_-agonists are among the most widely used agents in the treatment of patients with COPD and asthma [15, 26]. We found only 2 studies evaluating the effect of inhaled therapy containing inhaled beta-agonists on respiratory and cardiac function at rest in patients with COPD, but they were completed after days of therapy [15, 16]. Both studies attributed the increase in heart volume as well as increased vascularity as a beneficial response of the heart due to a decrease in the load imposed by the baseline hyperinflation of the thorax, once lung volumes decreased. However, resting VO_2_ reflecting peripheral oxygen uptake and respiratory function (minute ventilation) were not measured in either of the studies; therefore, it is possible that the increase in cardiac chamber size and output resulted from an adaptive response to the increased metabolic demand caused by the action of the beta-agonists on the muscle compartment. The improved cardiac function reported in those 2 studies may not be solely due to mechanical unloading of the heart with lung deflation; this is supported by the lack of relationship between improved lung function, including better IC, and the cardiac parameters in those reports [15, 16]. Consistent with those studies, we observed no relationship between improved lung mechanics and cardiac function. It remains possible that repeated doses of budesonide/formoterol may alter this acute response, as has been shown for 8 weeks of therapy with salbutamol [23].

This discussion is not meant to imply that decreasing lung volumes is not beneficial when they are the cause of poor cardiac function, as has been shown in over-ventilated patients with airflow limitations in the acute care setting [27, 28] and in patients who have undergone surgical or non-surgical lung volume reduction [13, 29, 30]. Interestingly, in contrast to our findings, the resting VO_2_ reported after surgical lung volume reduction is lower and not higher as we have shown in this report [29]. It could be argued whether an increase of 10 ml/min in oxygen uptake is clinically significant. However, patients with COPD spend over 80% of the day at rest, and this seemingly small difference per minute corresponds to 12.4 l of oxygen per day. Importantly, the increase in minute ventilation needed to match the increased oxygen demand observed was 0.862 l per minute. Over the 19 h patients with COPD would typically spend at rest, the daily increase in minute ventilation would be 982 l, a not insignificant amount in patients with a mean FEV_1_ of 1.46 l.

Perhaps the finding of an increased metabolic demand as a consequence of the beta-adrenergic effect may explain the weak relationship between the large improvement in lung function observed after maximal bronchodilation and the relatively small changes registered in the perception of dyspnea [31]. Indeed, in an older study with 2 doses of salmeterol, patients on the higher dose scored worse on the St. George’s Respiratory Questionnaire than those on the lower dose, even though the bronchodilation effect was significantly larger with the higher dose [32]. Taken together, these results suggest that increased work of breathing and metabolic demand could offset some of the relief a patient experiences from bronchodilation, causing some patients to continue to experience dyspnea despite improved spirometry values.

The current study has the advantages of the large number of observations (over 100 measurements for each treatment) and multicenter implementation (to mitigate center bias); however, there are also several limitations. First, direct cardiac function was not assessed using either central catheter or imaging. Notably, the measurement of metabolic parameters at rest and during steady-state conditions, and simultaneous measurement of respiratory variables provide important and novel information that is not readily available and that is less precise when measured during exercise. Second, intrathoracic pressures were not measured during the study, and thus it is possible that changes in such pressures caused some of the observed findings. However, the patients in the study were at rest, which is when intrathoracic swings show the lowest possible variations during tidal breathing [33, 34] and, in addition, the higher V_T_ observed with budesonide/formoterol compared with placebo minimizes this potential confounding factor. Third, the work of breathing was not measured directly, so it is not possible to discern whether the increased work observed is done by respiratory muscles or peripheral muscles. Importantly, the duty cycle was unchanged, which suggests that peripheral rather than respiratory muscles are performing this work; this phenomenon would need to be investigated further. Finally, the choice of medication (combination of ICS/LABA rather than LAMA or others) could be questioned, but it corresponds to the same class combination used in the report by Stone et al. [15] that we attempted to replicate.

## Conclusions

In summary, budesonide/formoterol via pMDI is a potent bronchodilator and lung “deflator” in patients with COPD and resting hyperinflation. At rest, there was a significant increase in metabolic and ventilatory demand after medication inhalation, as indicated by increased VO_2_, VCO_2_, and V_e_. These findings complement previous studies that suggested that lung deflation with inhaled bronchodilators improved cardiac function through improved respiratory mechanics. Studies are needed to clarify whether static lung deflation with medications given over longer periods of time increase cardiac function by improving cardio-ventilatory coupling or increasing peripheral oxygen demand. The exact balance between these mechanisms may help optimize medication use.

## Supplementary information

**Additional file 1.** Supplemental material.

## Data Availability

Data underlying the findings described in this manuscript may be obtained in accordance with AstraZeneca’s data sharing policy described at https://astrazenecagrouptrials.pharmacm.com/ST/Submission/Disclosure.

## Abbreviations

COPD: chronic obstructive pulmonary disease
FEV_1_: forced expiratory volume in 1 s
FVC: forced vital capacity
HR: heart rate
IC: inspiratory capacity
ICS: inhaled corticosteroid
IFR: inspiratory flow rate
LABA: long-acting beta_2_-agonist
LAMA: long-acting muscarinic antagonist
MRI: magnetic resonance imaging
pMDI: pressurized metered-dose inhaler
RR: respiratory rate
SVC: slow vital capacity
VCO_2_: carbon dioxide output
V_e_: minute ventilation
VO_2_: oxygen uptake
V_T_: tidal volume
