# Supplementary material for: Metabolic and cardiorespiratory effects of decreasing lung hyperinflation with budesonide/formoterol in COPD: a randomized, double-crossover, placebo-controlled, multicenter trial
Source: Respir Res. 2020 Jan 20;21:26. doi: 10.1186/s12931-020-1288-3 (PMC6972029; doi:10.1186/s12931-020-1288-3)
Supplement: Supplementary file 1 — Additional file 1. Supplemental material. [file 12931_2020_1288_MOESM1_ESM.docx]

**Additional file 1**

**Methods**

*Study design and patient selection*

This study was conducted at 5 study sites in the United States by IQVIA (Durham, NC, USA) from August 27, 2015 to August 12, 2016.

Eligible patients met the following inclusion criteria at screening: adult men or women aged 40 to 80 years (inclusive), clinical diagnosis of chronic obstructive pulmonary disease (COPD) according to the Global Initiative for Chronic Obstructive Lung Disease (GOLD) 2014 report [1] with a post-bronchodilator forced expiratory volume in 1 second (FEV_1_)/forced vital capacity (FVC) ratio <0.7, post-bronchodilator FEV_1_ ≤65% of the predicted value (using National Health and Nutrition Examination Survey [NHANES] predicted normal standards), increase in inspiratory capacity (IC) of >10% after the administration of 2 inhalations of open-label budesonide/formoterol via a pressurized metered-dose inhaler 160/4.5 µg (total dosage 320/9.0 µg), cigarette smoking history of >10 pack-years, signed the informed consent form prior to any study-specific procedures, and able to understand and comply with study requirements as judged by the investigator.

Exclusion criteria were as follows: employee or relative of an employee involved in the planning and/or conduct of the study; previous enrollment or randomization in the study; participation in another clinical study with any marketed or investigational biologic drug within 4 months or 5 half-lives of the previous drug (whichever was longer) prior to screening; unable to discontinue regular chronic COPD medications (including LAMAs, LABAs, and ICS) and/or unable or unwilling to comply with the study requirements; taking ultra–long-acting beta-agonists (uLABAs; indacaterol, vilanterol) or uLABA-containing products; taking phosphodiesterase type 4 inhibitors (roflumilast); taking oral corticosteroids on a chronic, regular basis; using daytime oxygen therapy; pregnant or breastfeeding; history of respiratory tract infection (including the upper respiratory tract) and/or pulmonary exacerbation within 6 weeks prior to screening; pulmonary resection or lung volume reduction surgery within 12 months prior to screening, or history of lung transplantation, or, in the investigator’s opinion, possibly in need of thoracotomy or other lung surgery during the study; history or current diagnosis of asthma and/or alpha-1 anti-trypsin deficiency (patients with a diagnosis of childhood asthma that had not required treatment after 12 years of age were eligible); known active tuberculosis; history of interstitial lung or massive pulmonary thromboembolic disease; history of bronchiectasis secondary to respiratory disease other than COPD; any clinically significant disease or disorder which, in the opinion of the investigator, might have put the patient at risk because of participation in the study, might have influenced the results of the study, or might have affected the patient’s ability to participate in the study; recent (within 12 months prior to screening) history of myocardial infarction, recent history of heart failure (New York Heart Association class III and IV [2]), pulmonary edema, and/or cardiac arrhythmia; previous or current history of lung cancer; history of cancer (within 5 years prior to screening), except for non-metastatic, non-melanoma skin cancer; unable to perform spirometry maneuvers or tolerate plethysmography; and known hypersensitivity to budesonide/formoterol, its monocomponents, or its excipients.

Use of tiotropium bromide was not allowed during the study duration; patients were given ipratropium bromide as a replacement therapy. All other LAMAs, LABAs, methylxanthines, and/or ICS could be used up to 48 hours prior to each study visit. Patients were prescribed a rescue medication (salbutamol [albuterol]; 1-2 inhalations 90 µg) for use as needed for COPD symptoms. Salbutamol and ipratropium bromide were withheld 12 hours before each visit.

Following washout of current medications, patients randomly received a single dose of 2 inhalations of budesonide/formoterol 160/4.5 μg or placebo with a BreatheRite^®^ spacer (medications were provided by AstraZeneca Pharmaceuticals LP, Wilmington, DE, USA). Patients were given unique randomization codes by investigators sequentially as they became eligible for randomization. Patients were assigned to treatment groups by a computer-generated random sequence using an interactive voice response system/interactive web response system. Study treatments were double-blinded for patients, investigators, and study personnel.

*Physiologic determinations*

Lung volumes were measured at screening using body plethysmography:

- Functional residual capacity (volume of gas present in the lung at end-expiration during tidal breathing)
- Thoracic gas volume (absolute volume of gas in the thorax at any point in time and any level of alveolar pressure)
- Total lung capacity (volume of gas in the lungs after maximal inspiration, or the sum of all volume compartments)
- Residual volume (volume of gas remaining in the lung after maximal exhalation [regardless of the lung volume at which exhalation was started])
- Slow vital capacity (SVC; maximal amount of air exhaled slowly from full inspiration to maximal expiration)

American Thoracic Society (ATS) and European Respiratory Society (ERS) guidelines were to be followed to provide accurate and comparable lung volume data. The body plethysmograph was configured to meet ATS/ERS recommendations for accuracy and precision [3].

Spirometry was performed at screening and at 60 minutes pre-dose and 60 minutes post-dose at each treatment visit. An electronic spirometer was to be used and the same apparatus used for each patient at each study center. Spirometry measured the following:

- IC (volume of air expressed in liters inspired when taking a slow full inspiration with no hesitation, from a position of passive end-tidal expiration to a position of maximum inspiration) via an SVC maneuver
- FVC (maximal volume of air exhaled with maximally forced expiratory effort from a position of maximal inspiration)
- FEV_1_ (volume of air expressed in liters exhaled during the first second of performance of the FVC)
- FEV_1_/FVC ratio

Efforts were made to perform all maneuvers approximately at the same temperature, approximately between 17°C and 34°C, and a log was maintained to document the maximum and minimum ambient temperature at least for those days when spirometric procedures were done.

The study center was responsible for calibrating and recording the calibration of the spirometer according to the recommendations of the manufacturer. Calibration was to be performed at least every day that the system was used by study patients, or after disassembling, cleaning and/or sensor replacement. In case of significant changes in temperature and/or barometric pressure within the same day, calibration was repeated before any other spirometry assessments were done. A log was maintained to document cleaning and calibration dates. All spirometry tests were printed, and an investigator signed and dated them.

The circumstances of patients’ tests were to be similar on all occasions with respect to time of the day and temperature as well as the technician, as much as possible. Measurements were to be made with the patient in a seated position and wearing a nose clip with no leaks between the mouth and mouthpiece.

The ATS and ERS guidelines were to be followed to provide accurate and comparable spirometric data. NHANES predicted normal standards were used for all patients. The spirometer was configured to meet ATS/ERS recommendations for accuracy and precision [4].

Gas exchange and respiratory variables were measured at screening and at 60 minutes pre-dose and 60 minutes post-dose at each treatment visit. A metabolic cart was used to measure the following and the same equipment was used for each patient at the study center:

- VO_2_ (oxygen uptake)
- VCO_2_ (carbon dioxide output)
- SaO_2_ (oxygen saturation)
- HR (heart rate)
- RR (respiratory rate)
- V_T_ (tidal volume)
- T_i_ (inspiratory time)
- T_e_ (expiratory time)

Non-invasive breath-to-breath analysis of oxygen uptake and carbon dioxide production were to be measured while the patient was seated and breathing through the mouthpiece with their nose clipped. In addition, continuous SaO_2_ was measured. All measurements were made at rest.

All continuous breath-to-breath variables were measured using the metabolic cart over a 5-minute period. Data collected over the first minute were discarded because placement of a mouthpiece will trigger reflex hyperventilation. The next 3 minutes provided the best results to be used for statistical analysis (assuming that the patient reached steady state, ie, there was no external interference such as cough, sneeze). If steady state was achieved, then the values obtained in 10-second epochs were used as raw data. On average, depending on the breathing rate, the recording provided approximately 18 epochs and the average of these measurements from each patient during each assessment (pre- and post-dose) was used for statistical analysis. This methodology avoids bias in selecting specific breaths or having 1 single value for the 3 minutes. If there were external interferences during the recording period, then the procedure was repeated once the patient returned to a normal steady state.

*Safety analyses*

The assessment of safety was based on the analyses of adverse events (AEs), vital signs, and electrocardiograms (ECG). All safety analyses were performed on the safety analysis set (ie, patients who received at least 1 dose of randomized study medication). No formal hypothesis testing of safety data was planned.

No laboratory safety assessments were conducted during the study. A comprehensive physical examination was performed by the principal investigator or a sub-investigator at screening. A 12-lead ECG was taken at screening and reported HR, P and QRS durations, and PR, QTcF, and QT intervals. Vital signs were taken prior to the ECG, with the patient at rest for a minimum of 2 minutes before assessment. ECG and vital sign measurements were to be completed before spirometry measurements.

All AEs were listed for patients in the safety analysis set. Overall AEs were tabulated (number and percentage of both patients and events) by treatment, system organ class, and preferred term. Separate summaries were provided for serious AEs (SAEs), AEs with outcome of death, AEs leading to discontinuation of study medication (DAEs), and common AEs. Listings of AEs included all AEs, SAEs, DAEs, and AEs leading to death. Listings included patients and treatment (received immediately prior to the AE). The timing of AEs was assigned to the period in which they first occurred, or worsened in intensity, relation or action taken with study medication. In the case of an AE worsening by 1 of these measures, it could be assigned to more than 1 period. The periods were prior and on-treatment. The on-treatment period was defined as any day between the date of first dose of study medication (Visit 2) to the date of follow-up Visit 6 inclusive, or date of withdrawal/discontinuation/last follow-up. Any AEs starting or worsening in this period were then considered a treatment-emergent AE. The prior period captured all AEs that first occurred prior to the first dose study medication (Visit 2).

*Statistical Analyses*

Sample size was determined using a similar pilot study [5]. Based on parameter estimates (mean placebo post-dose assessment in VO_2_; mean = 0.246 mL/min; standard deviation = 0.07 mL/min), sample sizes of 88, 40, and 24 patients were estimated to achieve at least 90% power to detect differences in pre-dose to post-dose change in VO_2_ between groups of 0.025 (10%), 0.037 (15%), and 0.049 (20%) mL/min, respectively, at the 5% 2-sided level of significance using a Student paired t-test and assuming a conservative intraclass correlation of 50%. Assuming a difference between groups of 15% and adding 10 extra patients (to account for the effect of patient dropouts and possible parameter differences with the reference study), a total sample size of 50 randomized patients was deemed sufficient to conduct this study.

Secondary efficacy measures included change from pre-dose to post-dose in oxygen pulse, gas exchange and cardiac parameters (VCO_2_, SaO_2_, and HR), spirometry (IC, FEV_1_, FVC, and FEV_1_/FVC), mean inspiratory flow, and MBS, and were analyzed in the same way as the primary endpoint.

**Supplementary References**

1. Global Initiative for Chronic Obstructive Lung Disease Inc. Global Strategy for the Diagnosis, Management and Prevention of COPD, Global Initiative for Chronic Obstructive Lung Disease (GOLD) 2014 Report. 2014.

2. The Criteria Committee of the New York Heart Association. Nomenclature and Criteria for Diagnosis of Diseases of the Heart and Great Vessels. 9th ed. Boston, MA: Little, Brown & Co; 1994.

3. Wanger J, Clausen JL, Coates A, et al. Standardisation of the measurement of lung volumes. Eur Respir J. 2005;26(3):511-522.

4. Miller MR, Hankinson J, Brusasco V, et al. Standardisation of spirometry. Eur Respir J. 2005;26(2):319-338.

5. Synn AJ, Pinto-Plata VM, Perham C, Celli BR, Divo M. Improvement in cost of breathing after use of budesonide/formoterol in COPD patients with static hyperinflation. Presented at American Thoracic Society (ATS) 2014 International Conference; May 16-21, 2014; San Diego, CA.

**Table S1. Independent Ethics Committees/Institutional Review Boards Consulted**

| **Center number** | **Name and address of IEC/IRB** | **Date of Approval** |
| --- | --- | --- |
| 7801 | Copernicus Group IRB  One Triangle Drive, Suite 100, PO Box 110605, Durham, NC 27709 | August 4, 2015 |
| 7802 | Copernicus Group IRB  One Triangle Drive, Suite 100, PO Box 110605, Durham, NC 27709 | August 4, 2015 |
| 7803 | Saint Francis Hospital and Medical Center IRB  114 Woodland Street, Hartford, CT 06105-1299 | October 21, 2015 |
| 7804 | Partners Human Research Committee/IRB  116 Huntington Avenue, Suite 1002, Boston, MA 02116 | February 9, 2016 |
| 7806 | Western IRB  1019 39th Avenue SE, Suite 120, Puyallup, PA 98374 | December 2, 2015 |

IEC, independent ethics committee; IRB, institutional review board.

**Table S2. Summary of AEs**

| **Category, n (%)** | **BUD/FORM**  **(N = 51)** | **Placebo  (N = 51)** |
| --- | --- | --- |
| Any AE | 13 (26) | 11 (22) |
| Any SAE (including death) | 0 | 1 (2) |
| Any AE leading to discontinuation of study drug | 0 | 2 (4) |
| Any causally related AE | 0 | 1 (2) |
| Most common AEs (≥3% of patients) |  |  |
| Dyspnea | 2 (4) | 2 (4) |
| COPD | 0 | 3 (6) |
| Back pain | 2 (4) | 0 |
| Nasopharyngitis | 2 (4) | 0 |

AEs, adverse events; BUD/FORM, budesonide formoterol; COPD, chronic obstructive pulmonary disease; SAE, serious adverse event.
